# Complete genome sequence of *Jonesia denitrificans* type strain (Prevot 55134^T^)

**DOI:** 10.4056/sigs.41646

**Published:** 2009-11-22

**Authors:** Rüdiger Pukall, Gabriele Gehrich-Schröter, Alla Lapidus, Matt Nolan, Tijana Glavina Del Rio, Susan Lucas, Feng Chen, Hope Tice, Sam Pitluck, Jan-Fang Cheng, Alex Copeland, Elizabeth Saunders, Thomas Brettin, John C. Detter, David Bruce, Lynne Goodwin, Amrita Pati, Natalia Ivanova, Konstantinos Mavromatis, Galina Ovchinnikova, Amy Chen, Krishna Palaniappan, Miriam Land, Loren Hauser, Yun-Juan Chang, Cynthia D. Jeffries, Patrick Chain, Markus Göker, Jim Bristow, Jonathan A. Eisen, Victor Markowitz, Philip Hugenholtz, Nikos C. Kyrpides, Hans-Peter Klenk, Cliff Han

**Affiliations:** 1 DSMZ - German Collection of Microorganisms and Cell Cultures GmbH, Braunschweig, Germany; 2 DOE Joint Genome Institute, Walnut Creek, California, USA; 3 Los Alamos National Laboratory, Bioscience Division, Los Alamos, New Mexico, USA; 4 Biological Data Management and Technology Center, Lawrence Berkeley National Laboratory, Berkeley, California, USA; 5 Oak Ridge National Laboratory, Oak Ridge, Tennessee, USA; 6 Lawrence Livermore National Laboratory, Livermore, California, USA; 7 University of California Davis Genome Center, Davis, California, USA

**Keywords:** *Actinobacteria, Actinomycetales*, *Micrococcineae*, *Jonesiaceae*, Gram-positive, irregular, nonsporulating rods, ox blood

## Abstract

*Jonesia denitrificans* (Prevot 1961) Rocourt *et al.* 1987 is the type species of the genus *Jonesia*, and is of phylogenetic interest because of its isolated location in the actinobacterial suborder *Micrococcineae*. *J. denitrificans* is characterized by a typical coryneform morphology and is able to form irregular nonsporulating rods showing branched and club-like forms. Coccoid cells occur in older cultures. *J. denitrificans* is classified as a pathogenic organism for animals (vertebrates). The type strain whose genome is described here was originally isolated from cooked ox blood. Here we describe the features of this organism, together with the complete genome sequence and annotation. This is the first completed genome sequence of a member of the genus for which a complete genome sequence is described. The 2,749,646 bp long genome with its 2558 protein-coding and 71 RNA genes is part of the *** G****enomic* *** E****ncyclopedia of* *** B****acteria and* *** A****rchaea * project.

## Introduction

Strain Prevot 55134^T^ (= DSM 20603 = ATCC 14870 = CIP 55.134) is the type strain of the species *Jonesia denitrificans*, the type species of the genus *Jonesia* [1]. The isolate originated from cooked ox blood [2]. *J*. *denitrificans* was originally placed into the genus *Listeria* and described as *L*. *denitrificans* by Prevot in 1961 [3], even though it differed morphologically from other members of the genus *Listeria.* Later, extensive analysis based on the determination of the G+C content [4], DNA-DNA hybridization [4], peptidoglycan type [5,6], as well as fatty acids and polar lipid pattern [5,7] confirmed the misclassification of the strain. As a consequence the strain was transferred to the new genus *Jonesia* by Rocourt *et al*. 1987 [1]. Five years later Stackebrandt and Prauser assigned *Jonesia* to the family *Cellulomonadaceae* despite being aware that *Jonesia* differed from members of other genera of the family *e.g.* in its G+C content, peptidoglycan type and its composition of isoprenoid quinones. The rationale to place *Jonesia* into this family was based on the finding that the 16S rRNA gene sequence analysis showed close relationship to *Promicromonospora* and *Cellulomonas* at that time [8]. Following extensive phylogenetic in 1995, studies *Jonesia* was subsequently excluded from the family *Cellulomonadacea* and placed in the family *Jonesiaceae,* within the suborder *Micrococcineae* [9]. With *Jonesia quinghaiensis*, an environmental isolate from mud of a soda lake in China, a second species of the genus was described by Schumann *et al.* in 2004 [10]. Two additional environmental strains closely related to J. denitrificans, with 98% and 99% 16S rRNA gene sequence similarity have been reported. These organisms were isolated from the microbial community of feed batch reactors for composting of household biowaste [11]. Here we present a summary classification and a set of features for *J. denitrificans* Prevot 55134^T^ together with the description of the complete genomic sequencing and annotation.

### Classification and features

**Figure 1:**
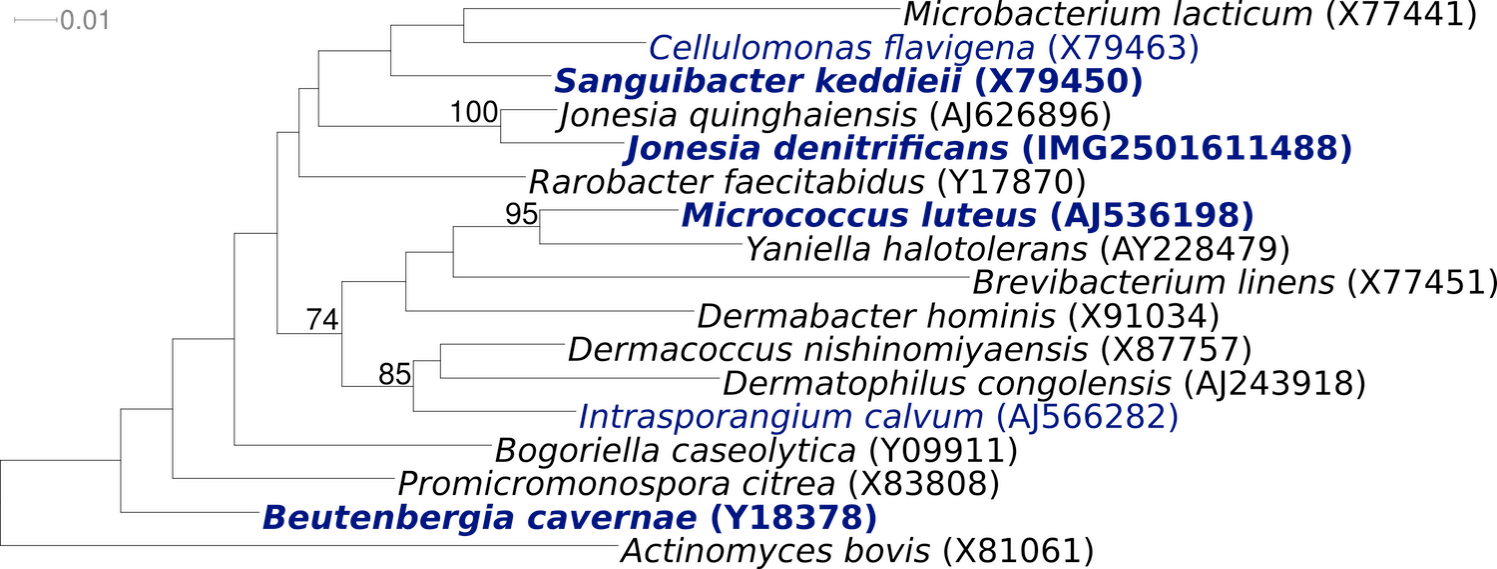
Phylogenetic tree highlighting the position of *J. denitrificans* Prevot 55134^T^ relative to all type strains of the genus *Jonesia* and the type strains of all families within suborder *Micrococcineae,* inferred from 1,417 aligned characters [19,20] of the 16S rRNA sequence under the maximum likelihood criterion [21]. Rooting was done with the type strain of *Actinomyces bovis*, the type species of *Actinomyces*, which is the type genus of the order *Actinomycetales*. The branches are scaled in terms of the expected number of substitutions per site. Numbers above branches are support values from 1,000 bootstrap replicates if larger than 60%. Lineages with type strain genome sequencing projects registered in GOLD [22] are shown in blue, published genomes in bold, *e.g.* the GEBA genomes *Beutenbergia cavernae* [23], and *Sanguibacter keddieii* [24].

Figure 1 shows the phylogenetic neighborhood of *J. denitrificans* strain Prevot 55134^T^ in a 16S rRNA based tree. The sequences of the five 16S rRNA gene copies in the genome of strain Prevot 55134^T^ do not differ from each other, and differ by eight nucleotides from the previously published 16S rRNA gene sequence of DSM 20603 (X78420).

**Figure 2:**
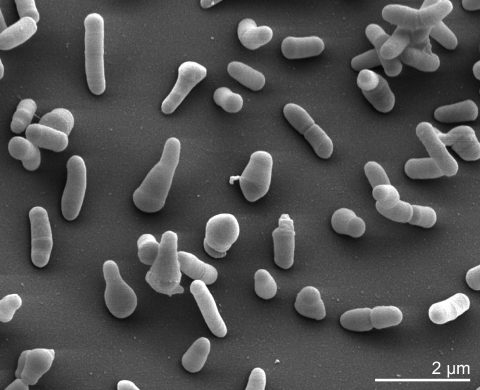
Scanning electron micrograph of *J. denitrificans* Prevot 55134^T^ (Manfred Rohde, Helmholtz Centre for Infection Research (HZI), Braunschweig)

*J. denitrificans* type strain cells are Gram-positive, typically coryneform and characterized by irregular rods, 0.3-0.5 µm in diameter and 2-3 µm in length. Coccoid forms occur in older cultures (Table 1),. No endospores are formed. (Figure 2). The cells are motile by means of peritrichous flagella (absent in Figure 2). Colonies range from 0.5 to 1.5 mm in diameter on BHI agar. The optimum temperature for growth is 30°C [16]. The organism is catalase positive, oxidase negative and utilizes D-cellobiose, D-galactose, D-sorbitol, turanose and acetic acid [10]. Cellulose, starch, DNA and RNA are hydrolyzed. In addition, *J. denitrificans* produces acid from a large variety of sugars, polysaccharides and other compounds as described by Seeliger and Jones in 1986 [16] and is capable of denitrification [25]. The natural habitat of the organism is not known, however, *J. denitrificans* is a known pathogen of rats and mice when injected intraperitoneally [16].

**Table 1:** Classification and general features of *J. denitrificans* Prevot 55134^T^ in accordance with the MIGS recommendations [12]

**MIGS ID**	**Property**	**Term**	**Evidence code**
		Domain *Bacteria*	TAS [13]
		Phylum *Firmicutes*	TAS [14]
		Class *Actinobacteria*	TAS [15]
		Subclass *Actinobacteridae*	TAS [15]
		Order *Actinomycetales*	TAS [15]
		Suborder *Micrococcineae*	TAS [15]
		Family *Jonesiaceae*	TAS [15]
		Genus *Jonesia*	TAS [1]
		Species *Jonesia denitrificans*	TAS [1]
		Type strain Prevot 55134	TAS [16]
	Gram stain	positive	TAS [16]
	Cell shape	irregular rods, coccoid	TAS [16]
	Motility	motile	TAS [16]
	Sporulation	nonsporulating	TAS [16]
	Temperature range	mesophile	TAS [16]
	Optimum temperature	30-37°C	TAS [16]
	Salinity	5% NaCl	TAS [16]
MIGS-22	Oxygen requirement	facultatively anaerobic	TAS [16]
	Carbon source	unknown	
	Energy source	unknown	
MIGS-6	Habitat	natural habitat not known	TAS [16]
MIGS-15	Biotic relationship	free living	NAS
MIGS-14	Pathogenicity	pathogenic for animals	TAS [16]
	Biosafety level	2	TAS [17]
	Isolation	cooked ox blood	TAS [2]
MIGS-4	Geographic location	France	NAS
MIGS-5	Sample collection time	not reported	
MIGS-4.1 MIGS-4.2	Latitude – Longitude	not reported	
MIGS-4.3	Depth	not reported	
MIGS-4.4	Altitude	not reported	

Evidence codes - IDA: Inferred from Direct Assay (first time in publication); TAS: Traceable Author Statement (i.e., a direct report exists in the literature); NAS: Non-traceable Author Statement (i.e., not directly observed for the living, isolated sample, but based on a generally accepted property for the species, or anecdotal evidence). These evidence codes are from the Gene Ontology project [18]. If the evidence code is IDA, then the property should have been directly observed for a living isolate by one of the authors, or an expert mentioned in the acknowledgements.

### Chemotaxonomy

The cell wall of strain Prevot 55134^T^ contains murein of type A4α, composed of L-Lys-L-Ser-D-Glu only [5,6], type A11.48 according to the DSMZ catalogue of strains (http://www.dsmz.de/microorganisms/main.php?content_id=35). In addition to the amino sugars muramic acid and glucosamine, galactosamine was detectable in the hydrolysate of the cell walls of *J. denitrificans* [5]. 12-Methyl-tetradonic acid (ai-C15:0) and hexadonic acid (C16:0) constituted the major cellular fatty acid, and minor amounts of 14-methyl-hexadonic acid (ai-C17:0) and tetradecanoic acid (C14:0) were also detected [6,7]. Diphosphatidylglycerol (DPG) and phosphatidylinositol (PI) were identified by TLC as the polar lipids [6] and menaquinone of the MK-9 type was detected as the major component.

## Genome sequencing information

### Genome project history

This organism was selected for sequencing on the basis of its phylogenetic position, and is part of the *** G****enomic* *** E****ncyclopedia of* *** B****acteria and* *** A****rchaea * project. The genome project is deposited in the Genomes OnLine Database [22] and the complete genome sequence in GenBank Sequencing, finishing and annotation were performed by the DOE Joint Genome Institute (JGI). A summary of the project information is shown in Table 2.

**Table 2:** Genome sequencing project information

**MIGS ID**	**Property**	**Term**
MIGS-31	Finishing quality	Finished
MIGS-28	Libraries used	Two Sanger libraries –8 kb pMCL200 and fosmid pcc1Fos
MIGS-29	Sequencing platforms	ABI3730
MIGS-31.2	Sequencing coverage	9.5x Sanger
MIGS-30	Assemblers	phrap
MIGS-32	Gene calling method	Prodigal, GenePRIMP
	INSDC ID	CP001706
	Genbank Date of Release	August 27, 2009
	GOLD ID	Gc01092
	NCBI project ID	20833
	Database: IMG-GEBA	2501533218
MIGS-13	Source material identifier	DSM 20603
	Project relevance	Tree of Life, GEBA

### Growth conditions and DNA isolation

*J. denitrificans* strain Prevot 55134^T^, DSM 20603, was grown in DSMZ medium 215 (BHI broth) [26], at 37°C. DNA was isolated from 0.5-1 g of cell paste using the JGI CTAB-Protocol with a modified protocol for cell lysis (ALM), according to Wu *et al*. [27].

### Genome sequencing and assembly

The genome was sequenced using only the Sanger platform. All general aspects of library construction and sequencing performed at the JGI can be found at the JGI website (http://www.jgi.doe.gov/). All reads were assembled using the phrap assembler. Possible mis-assemblies were corrected with Dupfinisher or transposon bombing of bridging clones [28]. Gaps between contigs were closed by editing in Consed, custom primer walk or PCR amplification. A total of 653 Sanger finishing reads were produced. The error rate of the completed genome sequence is less than 1 in 100,000. Together all sequenced reads provided 9.5x coverage of the genome. The final assembly consists of 35,028 Sanger 454 reads.

#### *Genome annotatio*n

Genes were identified using Prodigal [29] as part of the Oak Ridge National Laboratory genome annotation pipeline, followed by a round of manual curation using the JGI GenePRIMP pipeline (http://geneprimp.jgi-psf.org) [30]. The predicted CDSs were translated and used to search the National Center for Biotechnology Information (NCBI) non-redundant database, UniProt, TIGRFam, Pfam, PRIAM, KEGG, COG, and InterPro databases. Additional gene prediction analysis and functional annotation was performed within the Integrated Microbial Genomes Expert Review (IMG-ER) platform [31].

## Genome properties

**Figure 3:**
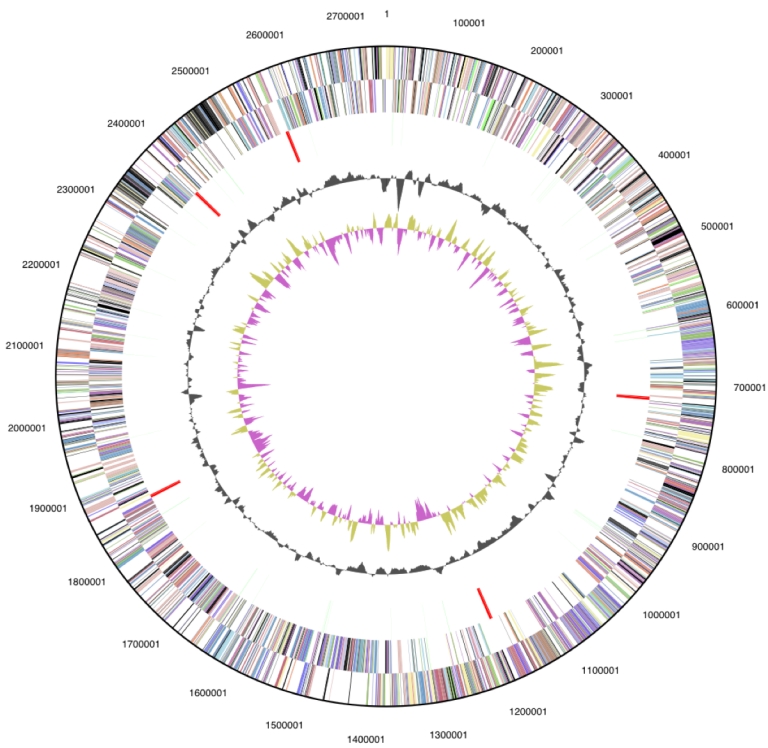
Graphical circular map of the genome. From outside to the center: Genes on forward strand (color by COG categories), Genes on reverse strand (color by COG categories), RNA genes (tRNAs green, rRNAs red, other RNAs black), GC content, GC skew.

The single replicon genome is 2,749,646 bp long with a 58.4% GC content (Table 3 and Figure 3). Of the 2,629 genes predicted, 2,558 were protein coding genes, and 71 RNAs. In addition, 47 pseudogenes were also identified. The majority of the genes (68.3%) were assigned with a putative function, while the remaining ones are annotated as hypothetical proteins. The distribution of genes into COGs functional categories is presented in Table 4.

**Table 3:** Genome Statistics

**Attribute**	Value	% of Total
Genome size (bp)	2,749,646	100.00%
DNA coding region (bp)	2,530,061	92.01%
DNA G+C content (bp)	1,606,252	58.42%
Number of replicons	1	
Extrachromosomal elements	0	
Total genes	2,629	100.00%
RNA genes	71	2.92%
rRNA operons	5	
Protein-coding genes	2,558	97.08%
Pseudo genes	47	1.78%
Genes with function prediction	1,796	68.31%
Genes in paralog clusters	223	8.46%
Genes assigned to COGs	1,830	69.45%
Genes assigned Pfam domains	1,849	70.17%
Genes with signal peptides	615	23.34%
Genes with transmembrane helices	682	25.88%
CRISPR repeats	0	

**Table 4:** Number of genes associated with the general COG functional categories

Code	value	%age	Description
J	144	5.6	Translation, ribosomal structure and biogenesis
A	1	0.0	RNA processing and modification
K	160	6.3	Transcription
L	100	3.9	Replication, recombination and repair
B	1	0.0	Chromatin structure and dynamics
D	22	0.9	Cell cycle control, mitosis and meiosis
Y	0	0.0	Nuclear structure
V	53	2.1	Defense mechanisms
T	89	3.5	Signal transduction mechanisms
M	93	3.6	Cell wall/membrane biogenesis
N	46	0.0	Cell motility
Z	0	0.0	Cytoskeleton
W	0	0.0	Extracellular structures
U	42	1.6	Intracellular trafficking and secretion
O	79	3.1	Posttranslational modification, protein turnover, chaperones
C	110	4.3	Energy production and conversion
G	210	8.2	Carbohydrate transport and metabolism
E	162	6.3	Amino acid transport and metabolism
F	68	2.7	Nucleotide transport and metabolism
H	93	3.6	Coenzyme transport and metabolism
I	54	1.1	Lipid transport and metabolism
P	121	4.7	Inorganic ion transport and metabolism
Q	23	0.9	Secondary metabolites biosynthesis, transport and catabolism
R	207	8.1	General function prediction only
S	141	5.5	Function unknown
-	728	28.5	Not in COGs
